# Supplementary material for: Localized spin-orbit polaron in magnetic Weyl semimetal Co3Sn2S2
Source: Nat Commun. 2020 Nov 5;11:5613. doi: 10.1038/s41467-020-19440-2 (PMC7644724; doi:10.1038/s41467-020-19440-2)
Supplement: Supplementary file 1 — Supplementary Information [file 41467_2020_19440_MOESM1_ESM.pdf]

Supplementary Figures for

Localized spin-orbit polaron in magnetic Weyl semimetal $Co_3Sn_2S_2$

Yuqing Xing[1,2,3†], Jianlei Shen[1,2†], Hui Chen[1,2,3†], Li Huang[1,2,3†], Yuxiang Gao[1,2,3], Qi Zheng[1,2,3], Yu-Yang Zhang[2,3], Geng Li[1,2,3], Bin Hu[1,2,3], Guojian Qian[1,2,3], Lu Cao[1,2,3], Xianli Zhang[1,2,3], Peng Fan[1,2,3], Ruisong Ma[1,2,3], Qi Wang[4], Qiangwei Yin[4], Hechang Lei[4], Wei Ji[4], Shixuan Du[1,2,3,5], Haitao Yang[1,2,3], Wenhong Wang[1,2,5], Chengmin Shen[1,2,3], Xiao Lin[2,1,3], Enke Liu[1,2,5], Baogen Shen[1,2,6], Ziqiang Wang[7*], and Hong-Jun Gao[1,2,3,5*]*

[1] *Beijing National Center for Condensed Matter Physics and Institute of Physics, Chinese Academy of Sciences, Beijing 100190, PR China*

[2] *School of Physical Sciences, University of Chinese Academy of Sciences, Beijing 100190, PR China*

[3] *CAS Center for Excellence in Topological Quantum Computation, University of Chinese Academy of Sciences, Beijing 100190, PR China*

[4] *Beijing Key Laboratory of Optoelectronic Functional Materials & Micro-Nano Devices, Department of Physics, Renmin University of China, Beijing 100872, PR China*

[5] *Songshan Lake Materials Laboratory, Dongguan, Guangdong 523808, PR China*

[6] *Institute of Rare Earths, Chinese Academy of Sciences, Jiangxi 341000, China*

[7] *Department of Physics, Boston College, Chestnut Hill, MA, USA*

†These authors contributed equally to this work

[*]Correspondence to: hjgao@iphy.ac.cn, wangzi@bc.edu, ekliu@iphy.ac.cn

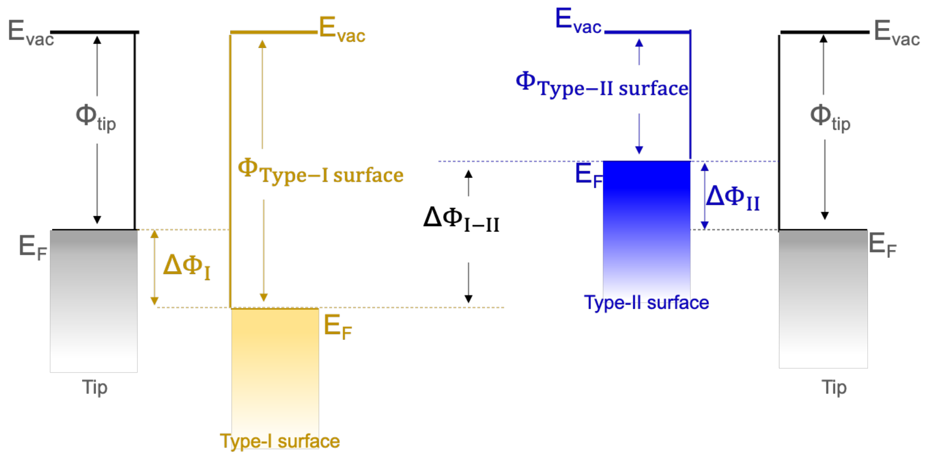

Supplementary Fig. 1 **Schematic of the relationship between work function and local contact potential difference (LCPD) (V_{CPD}) of the Type-I and the Type-II surfaces, respectively.** $V_{\mathrm{CPD}} = \frac{\Phi_{\mathrm{tip}} - \Phi_{\mathrm{sample}}}{-e}$. eV_{CPD} is the difference of the work function between the sample surface and tip. Φ_{tip} is the work function of the tip, $\Phi_{\mathrm{Type-I\ surface}}$ the work function of the Type-I surface, $\Phi_{\mathrm{Type-II\ surface}}$ the work function of the Type-II surface. Based on the nc-AFM measurements shown in the Fig. 1g of main text, we got: $\Delta\Phi_{\mathrm{I}}= \Phi_{\mathrm{tip}} - \Phi_{\mathrm{type-I\ surface}}$=-$eV_{CPD}^{I}$=0.22 eV; $\Delta\Phi_{\mathrm{II}}= \Phi_{\mathrm{tip}} - \Phi_{\mathrm{type-II\ surface}}$=-$eV_{CPD}^{II}$=-0.57 eV; Thus, $\Delta\Phi_{\mathrm{I-II}}= \Delta\Phi_{\mathrm{I}}$- $\Delta\Phi_{\mathrm{II}}$=0.79 eV.

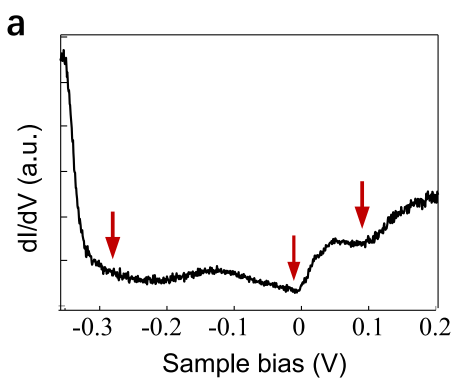

Supplementary Fig. 2 **Comparison between the experimental STS spectrum and theoretically calculated DOS projected onto the S surface. a** Typical dI/dV spectrum recorded on the Type-I surface using a W tip. **b** Total DOS projected onto the S surface (spin up + spin down). With a 0.35 eV energy offset, the experimental STS spectrum is highly consistent with the calculated DOS, and the main features (labeled by the arrows) are perfectly reproduced. It is worthy to mention that required offset of the Fermi energy in comparisons between DFT calculations and spectroscopic measurements on the surface is usually attributed to charge carrier induced chemical potential shift and/or correlation effects. A similar offset is also required to match the dI/dV data in a previous study of $Co_3Sn_2S_2$[1].

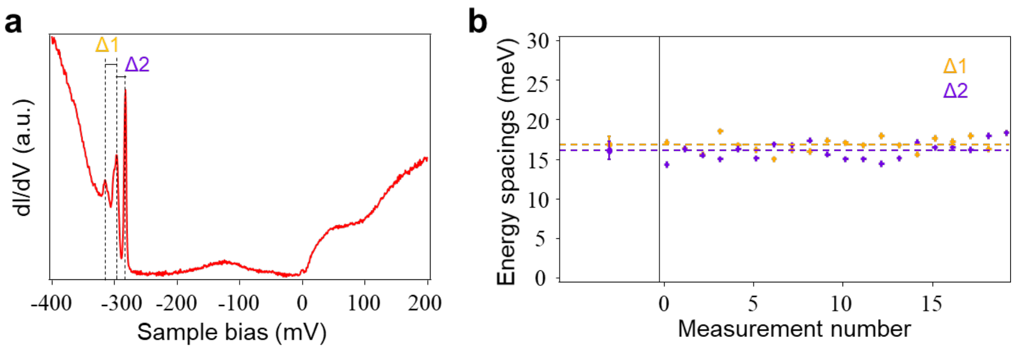

Supplementary Fig. 3 **Energy spacing of the bound states obtained at single S-vacancy. a** Typical dI/dV spectrum obtained at a single S-vacancy (V_s=-400 mV, I_t=500 pA, V_{mod}=0.5 mV), which is highly reproducible for different samples and STM tips. The energy spacings are labelled as Δ1 and Δ2 in (**a**). **b** Yellow and the purple dots in the left panel represent the average energy spacing of Δ1 and Δ2, respectively. All the individual reproducible measurements are displayed in the right panel, indicating that the peak energies are equally spaced.

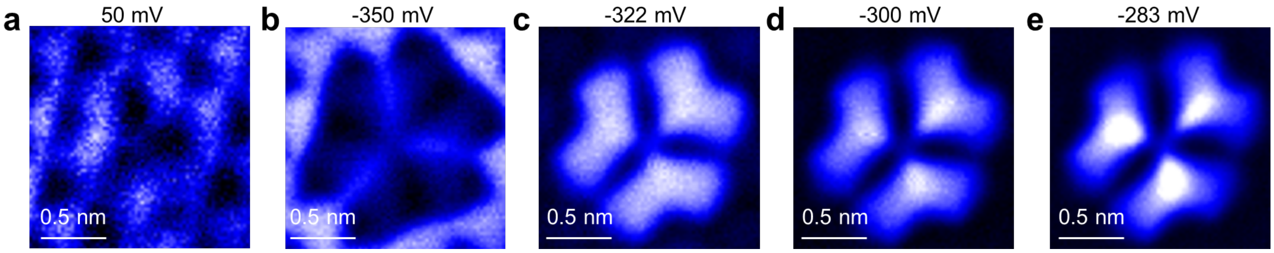

Supplementary Fig. 4 **dI/dV map in Fig. 2. a-e** dI/dV maps of single S-vacancy at different sample bias: 50 mV (**a**), -350 mV (**b**), -322 mV (**c**), -300 mV (**d**), and -283 mV (**e**), respectively.

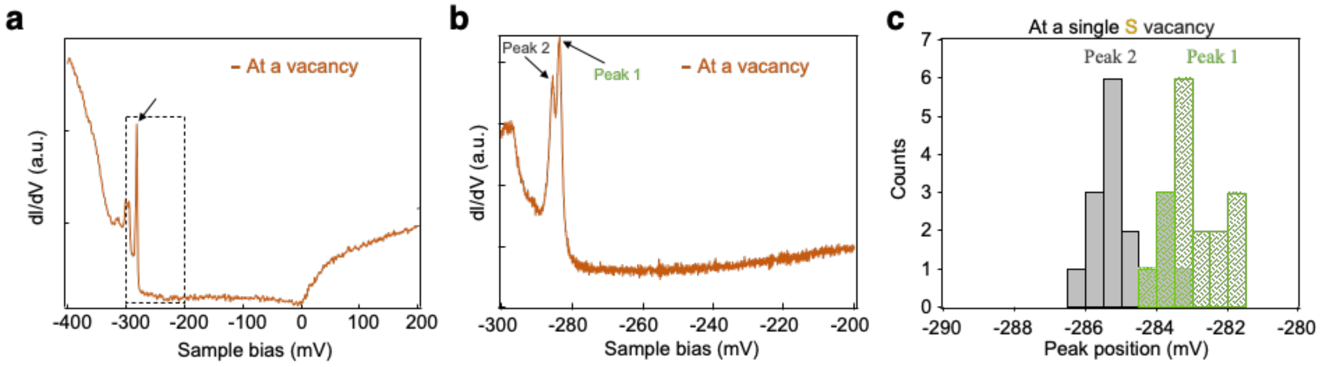

Supplementary Fig. 5 **Two-peak feature of dI/dV spectra obtained on a single S-vacancy site. a** dI/dV spectra at a single S-vacancy (V_s=-400 mV, I_t=500 pA, V_{mod}=0.5 mV). **b** Zoom-in of (**a**), ranging from -300 mV to -200 mV, showing a two-peak feature near -283 mV (V_s=-300 mV, I_t=500 pA, V_{mod}=0.5 mV), possibly due to the exchange field induced energy splitting. **c** Two sets of energy peaks obtained from 17 different single-S vacancies show a similar variation of ±1.5 mV, indicating that the bound states are robust at the single S-vacancy sites.

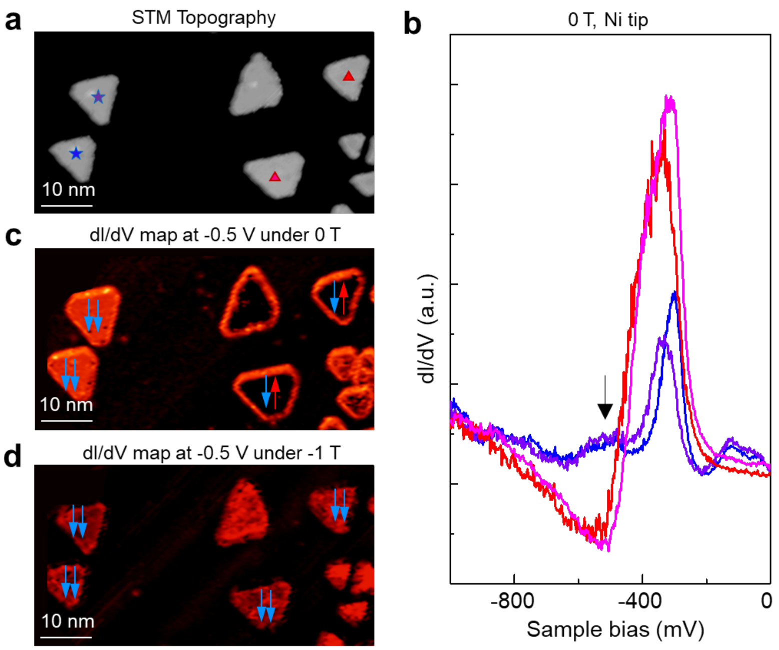

Supplementary Fig. 6 **Calibration of spin-polarized Ni tip on Co/Cu(111). a** STM image, showing the topography of Co islands on Cu(111). (V_s=-400 mV, I_t=100 pA). **b** dI/dV spectra obtained on four Co islands with the same stack configurations using the Ni tip. The blue and purple curves are obtained on the blue and purple stars in (**a**), the pink and red curve are obtained on pink and red triangles in (**a**). **c-d** dI/dV map of (**a**) at the energy of -0.5 V at magnetic field of 0 T (**c**) and -1 T (**d**). The contrast on the same stacked islands at 0 T and the absence of contrast at -1 T confirm the spin-polarization of the Ni tip[2,3]. The spin orientations of the Co island and tip are labelled by the upward (red) or downward (blue) arrows.

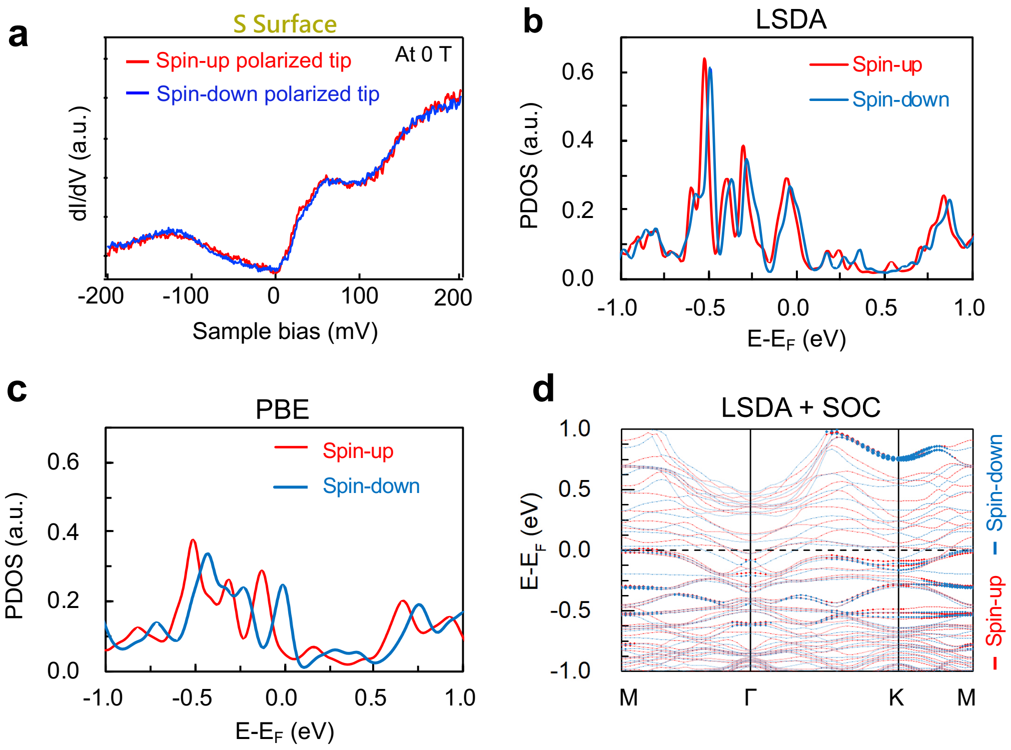

Supplementary Fig. 7 **Spin-polarized dI/dV spectra and calculated PDOS of the S-terminated surface.** **a** Spin polarized dI/dV spectrum of the S-terminated surface (same as Fig. 3a). **b** Spin-resolved density of state (DOS) projected on a surface S atom of the S-terminated surface with LSDA functional. The DOS curves of the two spins of S surface are almost degenerate. The net magnetic moment of the S atom is 0.003 μB**.** **c** Spin-resolved density of state (DOS) projected on a surface S atom of the S-terminated surface with PBE functional. The DOS curves of the two spins of S surface show similar shapes but with a ~ 0.2 eV energy shift. The net magnetic moment of the S atom is 0.005 μ_B. **d** The energy bands projected on the S surface calculated with LSDA functional and spin-orbital coupling.

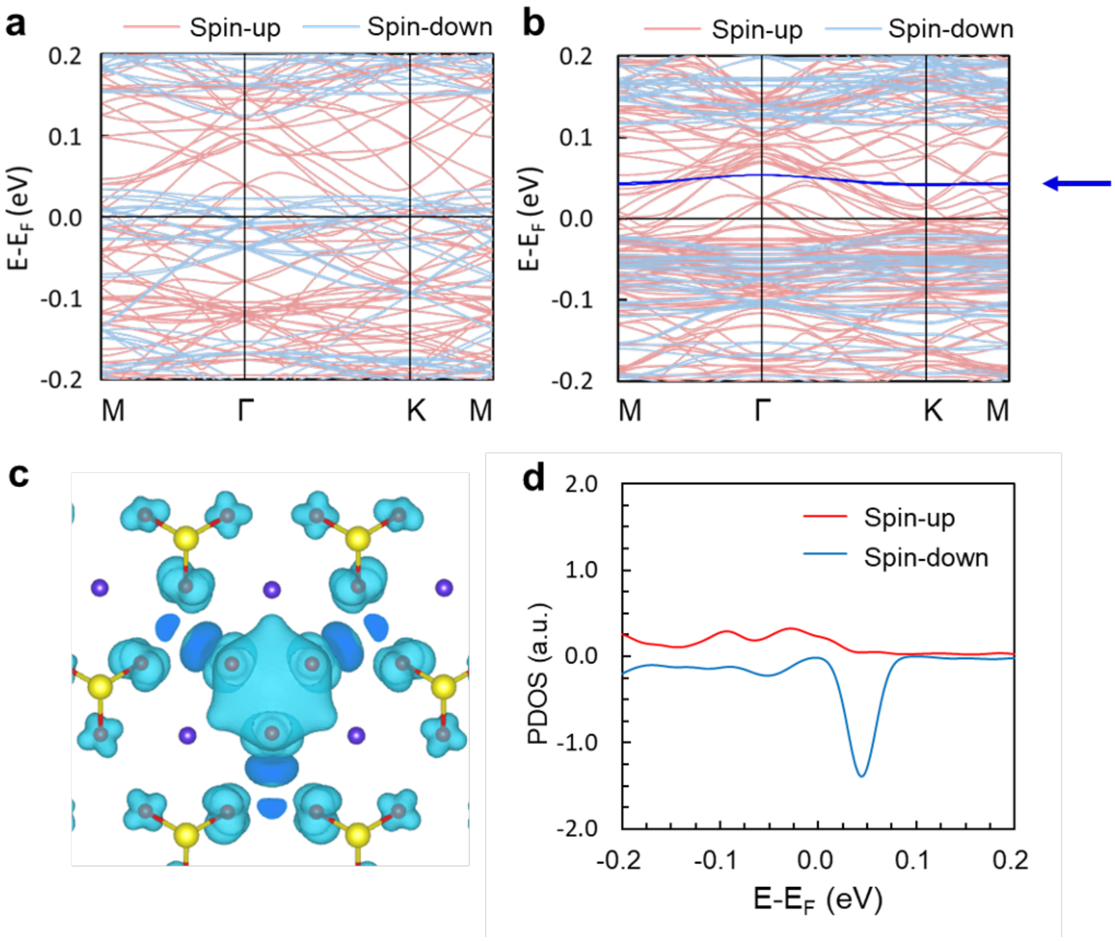

Supplementary Fig. 8 **Calculated electronic structures of the pristine and defective (with a S vacancy) S-terminated surfaces.** Band structures of the pristine (**a**) and the defective (**b**) S surface (in a 4 × 4 supercell). By comparing (**a**) and (**b**), a bound state is clearly visible as marked by the blue solid line and labelled by the blue arrow in (**b**). The overall line shape and features of the DFT calculations reasonably agrees with those of the experiment, with an overall shift of about 0.35 eV to the Fermi energy (the energy shift has been discussed in Supplementary Fig. 2b). It's worthy to point out that there is a ~0.1 eV gap in the spin-down channel. This band gap is reflected as a dip feature in Supplementary Fig. 7c, which is due to the 0.05 eV smearing width used in plotting the figure. **c** Spatial distribution of the wavefunction norm of the bound state marked in (**b**), which confirms its bound state nature. An isosurface of 3.0×10^{-4} e/Bohr^3 is used. **d** Spin-resolved DOS projected on the S vacancy, showing appreciable spin-polarization, in agreement with the experimental observation. We want to point out that the bound state is almost two-fold degenerated, while there are at least three bound states observed experimentally. The experiment-theory discrepancy reflects the limitation of standard DFT to explain the defect excitations in magnetic Weyl semimetals. The magnetic moment integrated within the sphere of radius 1.164 Å (the Wigner-Seitz

radius of S atom) is 0.04 μ_B. The experimental data (Fig. 2d-h) indicate that the size of the localized polaron is much larger and contains a cluster of atoms around the S vacancy. Therefore we estimate the contribution to the magnetic moment of the localized polaron from one S vacancy and three neighboring Co atoms. The total pseudospin (electron spin + atomic orbital) magnetic moment from the calculation is 0.85 μ_B. For the three Co atoms nearest to S vacancy, the orbital moment is 0.09 μ_B and the total magnetic moment is 0.81 μ_B. In the standard formula of DFT for non-collinear spin calculations, the diamagnetic term of the Dirac equation is ignored, which cannot account for the diamagnetic orbital magnetization contribution. This paramagnetic spin-orbit moment follows the magnetic field direction and cannot explain the observed net diamagnetic moment of 1.35 μ_B that always points in the opposite direction of the magnetic field. The exact diamagnetic orbital moment thus equals to the value of the net diamagnetic moment plus that of the paramagnetic moment revealed by DFT, i.e. being of the order ~ (1.35 + 0.85) =2.20 μ_B. The discrepancy on diamagnetic moment between our experiment and DFT calculation, indeed, supports the spin-orbit nature of the found polaron.

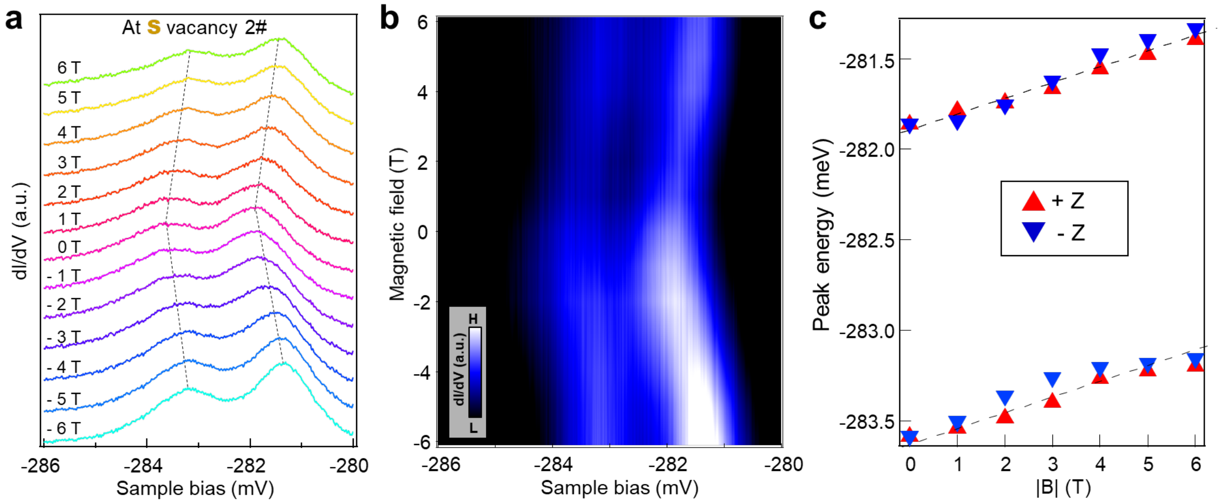

Supplementary Fig. 9 **Anomalous Zeeman shift of the SOP under external magnetic fields.** This set of data is taken on another single S-vacancy, showing the reproducibility of the results in Fig. 4a-c. **a** Magnetic field dependence of the peak positions on a single S-vacancy (V_s=-400 mV, I_t=500 pA, V_{mod}=0.5 mV). **b** Intensity plot of the interpolated field-dependent spectra, showing a similar shift as that in (**a**). **c** Peak energy as a function of the absolute value of the external magnetic field in (**a**).

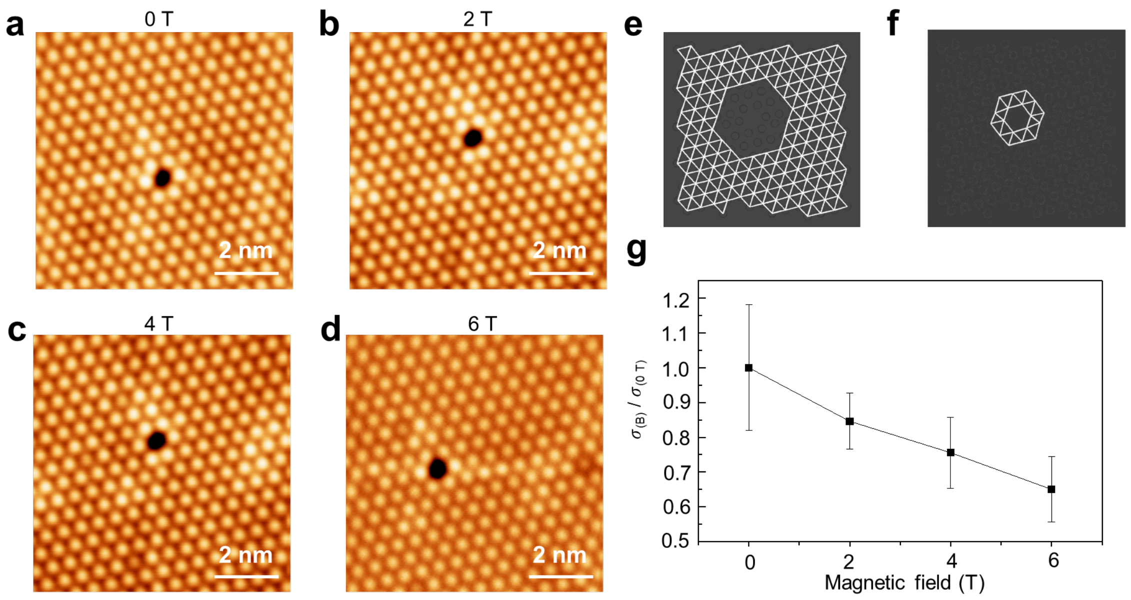

Supplementary Fig. 10 **Statistical analysis of magnetic-field-dependent local atomic displacement induced by a single S-vacancy. a-d** STM images of a single S-vacancy obtained under 0 T (**a**), 2 T (**b**), 4 T (**c**), and 6 T (**d**), respectively (V_s=-400 mV, I_t=100 pA). **e** An example of extracted nearest neighbor distances in the region far away from the vacancy in (**c**). The average distance is regarded as the standard atom-atom distance $\bar{d}$. **f** An example of extracted nearest neighbor distances (d_i, i=1, 2, 3…, 36) in the region of the vacancy in (**c**), according to the dI/dV map of vacancy in Fig. 2h in the main text. The center position of single atom was obtained by fitted the STM image with bivariate normal distribution:

$$f(x,y) = C_1 + \frac{C_2}{2\pi\sigma_X\sigma_Y\sqrt{1-\rho^2}}\exp\left(-\frac{1}{2(1-\rho^2)}\left[\frac{(x-\mu_X)^2}{\sigma_X^2} + \frac{(y-\mu_Y)^2}{\sigma_Y^2} - \frac{2\rho(x-\mu_X)(y-\mu_Y)}{\sigma_X\sigma_Y}\right]\right)$$

Where C_1 and C_2 are fitting constants, σ_X and σ_Y are the standard deviation of the distribution along x and y direction, respectively, ρ is the correlation between x and y direction, and (μ_X, μ_Y) is the expectation of the distribution, i.e. the position of atom. The local atomic displacement of a single S-vacancy σ is defined as $\sigma = \sqrt{\sum_{i=1}^{36} \frac{(d_i-\bar{d})^2}{36\bar{d}}}$. **g** The local atomic displacement as a function of the magnetic field strength, showing that the displacement decreases with increasing magnetic field. The error bars reflect the variation in the determination of the atomic position and in the calculation of the nearest neighbor atom-atom distances.

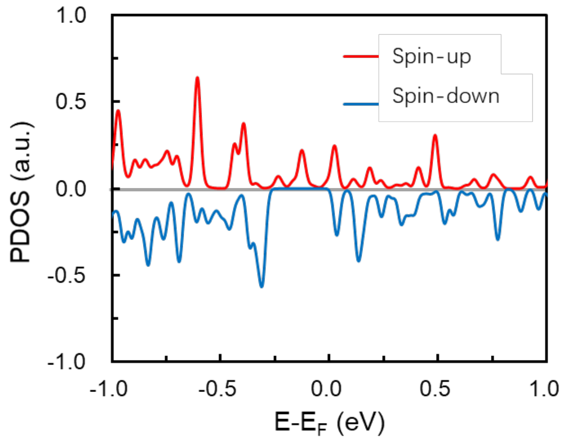

Supplementary Fig. 11 **Density of states projected onto a S vacancy site in a (2×2×2) bulk supercell.** It is clearly shown that the S vacancy in bulk $Co_3Sn_2S_2$ is magnetic. The magnetic moment integrated within a S vacancy site is 0.04 μ_B. The existence of S vacancies in bulk also increases the magnetic moment of each nearby Co atom from 0.35 μ_B to 0.72 μ_B.

Supplementary References:

1. Morali, N. *et al.* Fermi-arc diversity on surface terminations of the magnetic Weyl semimetal $Co_3Sn_2S_2$. *Science* **365**, 1286–1291 (2019).
2. Wiesendanger, R. Spin mapping at the nanoscale and atomic scale. *Rev. Mod. Phys.* **81**, 1495–1550 (2009).
3. Pietzsch, O., Kubetzka, A., Bode, M. & Wiesendanger, R. Spin-polarized scanning tunneling spectroscopy of nanoscale cobalt Islands on Cu(111). *Phys. Rev. Lett.* **92**, 057202 (2004).
